# Parasites of parasites of bats: Laboulbeniales (Fungi: Ascomycota) on bat flies (Diptera: Nycteribiidae) in central Europe

**DOI:** 10.1186/s13071-017-2022-y

**Published:** 2017-02-21

**Authors:** Danny Haelewaters, Walter P. Pfliegler, Tamara Szentiványi, Mihály Földvári, Attila D. Sándor, Levente Barti, Jasmin J. Camacho, Gerrit Gort, Péter Estók, Thomas Hiller, Carl W. Dick, Donald H. Pfister

**Affiliations:** 1 Department of Organismic and Evolutionary Biology, Harvard University, Cambridge, Massachusetts USA; 2 Department of Biotechnology and Microbiology, University of Debrecen, Debrecen, Hungary; 3 Department of Evolutionary Zoology and Human Biology, University of Debrecen, Debrecen, Hungary; 4 Department of Ecology and Evolution, University of Lausanne, Lausanne, Switzerland; 5 Museum of Zoology, Lausanne, Switzerland; 6 Department of Parasitology and Parasitic Diseases, University of Agricultural Sciences and Veterinary Medicine, Cluj-Napoca, Romania; 7 Romanian Bat Protection Association, Satu Mare, Romania; 8 Biometris, Wageningen University, Wageningen, The Netherlands; 9 Department of Zoology, Eszterházy Károly University, Eger, Hungary; 10 Institute of Evolutionary Ecology and Conservation Genomics, University of Ulm, Ulm, Germany; 11 Department of Biology, Western Kentucky University, Bowling Green, Kentucky USA

**Keywords:** *Arthrorhynchus*, Bat flies, Ecological specificity, Ectoparasitic fungi, Host specificity, Hyperparasitism

## Abstract

**Background:**

Bat flies (Streblidae and Nycteribiidae) are among the most specialized families of the order Diptera. Members of these two related families have an obligate ectoparasitic lifestyle on bats, and they are known disease vectors for their hosts. However, bat flies have their own ectoparasites: fungi of the order Laboulbeniales. In Europe, members of the Nycteribiidae are parasitized by four species belonging to the genus *Arthrorhynchus*. We carried out a systematic survey of the distribution and fungus-bat fly associations of the genus in central Europe (Hungary, Romania).

**Results:**

We encountered the bat fly *Nycteribia pedicularia* and the fungus *Arthrorhynchus eucampsipodae* as new country records for Hungary. The following bat-bat fly associations are for the first time reported: *Nycteribia kolenatii* on *Miniopterus schreibersii*, *Myotis blythii*, *Myotis capaccinii* and *Rhinolophus ferrumequinum*; *Penicillidia conspicua* on *Myotis daubentonii*; and *Phthiridium biarticulatum* on *Myotis capaccinii*. Laboulbeniales infections were found on 45 of 1,494 screened bat flies (3.0%). We report two fungal species: *Arthrorhynchus eucampsipodae* on *Nycteribia schmidlii*, and *A. nycteribiae* on *N. schmidlii*, *Penicillidia conspicua*, and *P. dufourii. Penicillidia conspicua* was infected with Laboulbeniales most frequently (25%, *n* = 152), followed by *N. schmidlii* (3.1%, *n* = 159) and *P. dufourii* (2.0%, *n* = 102). Laboulbeniales seem to prefer female bat fly hosts to males. We think this might be due to a combination of factors: female bat flies have a longer life span, while during pregnancy female bat flies are significantly larger than males and accumulate an excess of fat reserves. Finally, ribosomal DNA sequences for *A. nycteribiae* are presented.

**Conclusions:**

We screened ectoparasitic bat flies from Hungary and Romania for the presence of ectoparasitic Laboulbeniales fungi. *Arthrorhynchus eucampsipodae* and *A. nycteribiae* were found on three species of bat flies. This study extends geographical and host ranges of both bat flies and Laboulbeniales fungi. The sequence data generated in this work contribute to molecular phylogenetic studies of the order Laboulbeniales. Our survey shows a complex network of bats, bat flies and Laboulbeniales fungi, of which the hyperparasitic fungi are rare and species-poor. Their host insects, on the other hand, are relatively abundant and diverse.

**Electronic supplementary material:**

The online version of this article (doi:10.1186/s13071-017-2022-y) contains supplementary material, which is available to authorized users.

## Background

### Bats and bat flies

Bats (Mammalia: Chiroptera) contain more than 1,300 described species and are the second-most diverse group of living mammals, after rodents [1]. The lineage has evolved numerous adaptations that uniquely and effectively expand their ecological amplitude. These include flight, echolocation and a generally nocturnal lifestyle [2]. Moreover, bats are capable of utilizing a wide variety of food sources including insects, other vertebrates, blood, fruit and nectar. Bats vary greatly in their sociality ranging from solitary to the largest congregations of mammals numbering tens of millions of individuals. Finally, they vary in the roosts they use during day and night, from large and permanent structures such as caves and mines, to intermediate structures such as buildings and hollow trees, to ephemeral structures such as leaf tents and plant foliage [3]. Owing in part to these unique adaptations, bats are also parasitized by a plethora of arthropod lineages, including mites, ticks, bugs, fleas and flies. Among these parasites, the flies (Diptera: Hippoboscoidea: Streblidae and Nycteribiidae) are among the most conspicuous. Commonly known as “bat flies”, these specialized insects are known only from bats where they live in the fur and on the flight membranes and feed on blood [4]. Bat flies currently are divided into two families. The family Streblidae contains about 230 described species, which are cosmopolitan but generally richer in the Western Hemisphere. The family Nycteribiidae contains about 275 species, also occurring worldwide but generally more speciose in the Eastern Hemisphere [5]. Both families are considered tropical or subtropical in distribution, though certain species occur at northern or southern latitudes.

The flies are “semi-permanent” parasites with adaptations that help keep them associated with their bat hosts. Adult females rear three larval stages internally, nourished by milk glands, and adults of both sexes generally do not often leave the host bats. One exception are gravid females, who temporarily leave their host to deposit the terminal third-instar larvae on the roost substrate. Here, the larva soon forms a puparium and at approximately three weeks development the new adult emerges to seek a host bat in order to feed and find mates [4]. However, some studies [6] suggest that the duration of European bat fly pupal development is dependent in part upon the presence of bats in the roost. Thus, although larval stages are telescoped and tied to the host *via* the female fly, there is necessarily a period of off-host development that presents opportunities for flies to interact with other fly and host species inside the roost, and with the roosting environment itself [7]. Despite opportunity to colonize multiple host species, the emerging consensus is that bat flies are quite specific to individual host species, at least in ecological time. Although some experimental work has evaluated host choice and specificity [8, 9], much of our understanding of host specificity comes from large surveys of bats and parasites. When care is taken in the handling of bats and bat flies in the field, a remarkably high degree of host specificity is noted [10]. In particular, bat social structure, the roost environment, and species fidelity to roosting structures play key roles in parasite dynamics [11, 12]. Bat species that live in large groups and roost in large and long-lived structures that they return to with high fidelity increases parasitism generally, which in turn should raise transmission frequency and opportunities for selection toward parasitism in organisms associated with bats and with bat parasites.

### Laboulbeniales

Laboulbeniales (Fungi: Ascomycota) are microscopic parasites of myriad arthropod hosts. They grow onto the integument of three subphyla within Arthropoda: Chelicerata (infecting only mites), Myriapoda (millipedes), and Hexapoda (insects) [13]. Unlike other multicellular fungi, Laboulbeniales do not grow mycelia or hyphae. Instead they form a *thallus* (plural: *thalli*), which is derived from the enlargement and subsequent divisions of a single two-celled ascospore. A thallus typically consists of three major parts: a multicellular receptacle, which attaches to the host through the (often blackened—melanized) foot or root-shaped haustorium that may penetrate the body of the host; a single or multiple perithecia, the spore-forming structure(s); and appendages bearing antheridia, which produce spermatia.

While about 80% of the currently described species of Laboulbeniales are found on Coleoptera, only 10% parasitize Diptera [14]. Laboulbeniales parasitizing flies belong to eight genera: *Arthrorhynchus* Kolenati, *Dimeromyces* Thaxt., *Gloeandromyces* Thaxt., *Ilytheomyces* Thaxt., *Laboulbenia* Mont. & C.P. Robin, *Nycteromyces* Thaxt., *Rhizomyces* Thaxt. and *Stigmatomyces* H. Karst. The genus *Laboulbenia* is by far the largest genus with close to 600 recognized species with a wide array of hosts, and only 24 of those are known from flies [15]. *Stigmatomyces* is the second-largest genus in the order, with 144 described species, all of which occur on flies [16]. The genera *Arthrorhynchus*, *Gloeandromyces* and *Nycteromyces* are specific to bat flies, while all other genera have never been recorded from these two host families.

*Arthrorhynchus* is apparently restricted to Eastern Hemisphere species of the Nycteribiidae, which are also most diverse in the Eastern Hemisphere. Thaxter [17] recognized three species: *A. cyclopodiae* Thaxt., *A. eucampsipodae* Thaxt. and *A. nycteribiae* (Peyr.) Thaxt. Two additional species, *A. diesingii* and *A. westrumbii*, were described in the 1850s [18], but were soon synonymized with *Arthrorhynchus nycteribiae* (as *Helminthophana*) [19]. Merola [20] described a fourth species, *A. acrandros*. This species, however, is very similar to *A. nycteribidae* and likely represents the same species. New collections are needed before making a final decision about synonymy because Merola’s original material is likely lost (W. Rossi personal communication). Both species *A. acrandros* (Italy) and *A. cyclopodiae* (Papua New Guinea) are only known from the type collection.

A comprehensive study was conducted by Blackwell [21] to screen nycteribiid bat flies for presence of *Arthrorhynchus* spp. She screened 2,517 individuals, of which 56 were infected by *A. eucampsipodae* or *A. nycteribiae*, denoting an infection prevalence of 2.2% [21].

**Fig. 1:**
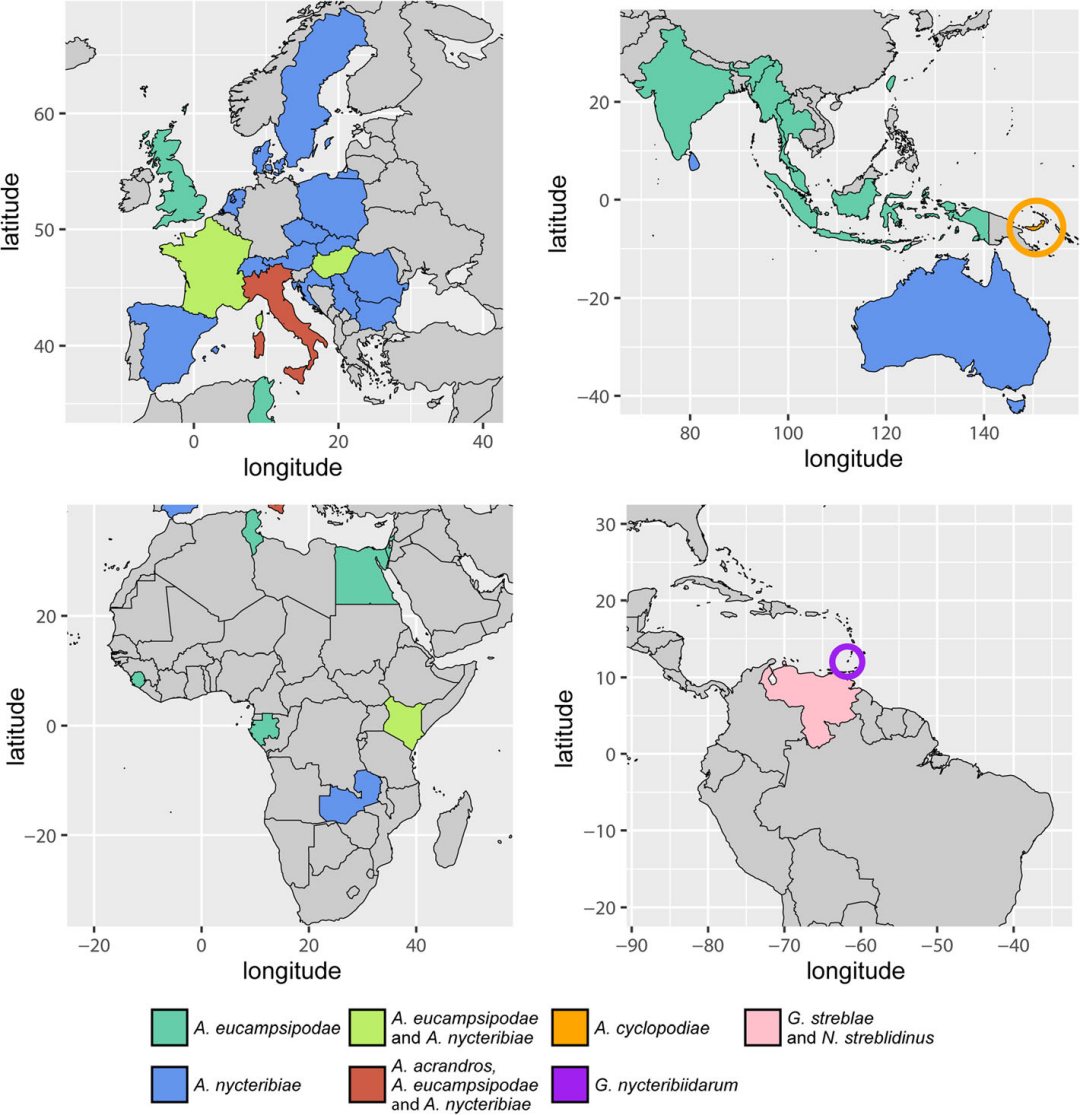
Review of all bat fly-associated Laboulbeniales. Global distribution of bat fly-Laboulbeniales interactions (up to November 2016). All published records from the literature are incorporated in this figure, as well as the reports from the present paper. For a detailed overview of literature records, see Additional file 1: Table S1

The diversity of the other two bat fly infecting genera is restricted, as is knowledge of their distribution and biology. Two species of *Gloeandromyces* are known: *G. nycteribiidarum* (Thaxt.) Thaxt. and *G. streblae* Thaxt. Both species have been reported only once [22], although several aspects of the biology of their hosts have recently been the focus of different studies. *Gloeandromyces nycteribiidarum* was described on *Megistopoda aranea* (Coquillett, 1899) (as *Pterellipsis aranea*) (Streblidae) from Grenada, *G. streblae* on *Strebla wiedemanni* Kolenati, 1856 (as *S. vespertilionis*) (Streblidae) from Venezuela. *Nycteromyces* is monotypic. Its single species, *N. streblidinus* Thaxt., was described on *Strebla wiedemanni* (as *S. vespertilionis*) from Venezuela [23] and has not been reported since. Interestingly, *G. streblae* and *N. streblidinus* were described from the same bat fly specimen (“No. 2073/a,” deposited at the Harvard Museum of Comparative Zoology). Both *Gloeandromyces* and *Nycteromyces* seem restricted in distribution to the Western Hemisphere, where they are associated with host species of the Western Hemisphere clade of the paraphyletic Streblidae [24]. The currently known distributional records, fungus-bat fly and fungus-bat associations are summarized in Fig. 1 and Additional file 1: Table S1. From the two countries considered in this paper, only *Arthrorhynchus nycteribiae* (Peyr.) Thaxt. is reported, from the nycteribiid *Penicillidia conspicua* Speiser, 1901.

Recently, studies on the distribution, population dynamics, host-choice and host-parasite interactions of different Laboulbeniales enabled assessment of the biology of this peculiar fungal group [25–27]. Such studies have successfully focused on locally abundant host insects forming colonies or aggregates (ants and ladybirds). Since bat flies are easily collected during bat surveys and are restricted to microhabitats where they may reach high population densities, they are interesting research targets for similar Laboulbeniales-oriented studies. With this collaborative project we aim to contribute to the research of parasitism of bat flies by Laboulbeniales fungi. The current scantiness of material precludes hypotheses about parasitism, host specificity, etc. As for five species of Laboulbeniales from bat flies only the type collection exists - *A. acrandros*, *A. cyclopodiae*, *G. nycteribiidarum*, *G. streblae* and *N. streblidinus - *our first effort has been to collect bat flies and screen these and existing bat fly collections for the presence of Laboulbeniales. Despite having many features that contribute to the difficulties in studying the Laboulbeniales [28], they do remain intact on dead host individuals. This way, historical collections of hosts can be used to record parasite prevalence, host specificity and population dynamics through time [13].

Here we extend geographical and host ranges, and discuss host associations. Our results also illustrate how even decade-old insect collections (with detailed collection data) can be used to uncover new host-parasite networks and the underlying factors.

## Methods

### Sampling sites

**Fig. 2:**
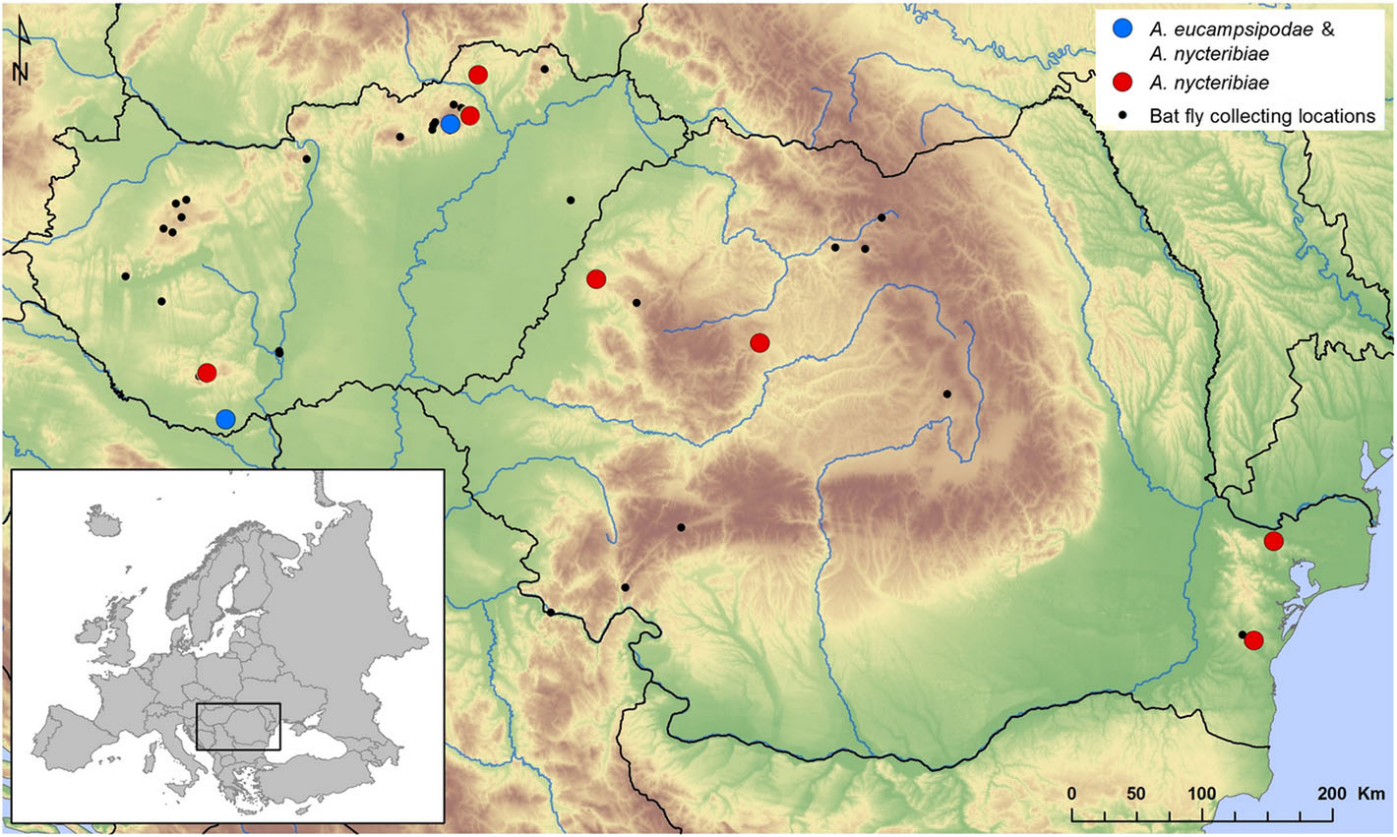
Geographical map of the studied area. Shown are all sampled locations, with indication of those locations where we found infected bat flies. *Arthrorhynchus eucampsipodae* is reported only from Hungary, while *A. nycteribiae* is reported in both Hungary and Romania. Black dots represent locations where no Laboulbeniales fungi were found on the collected bat flies

Bat flies were collected during bat surveys in the Romanian Carpathians and the Dobrogean Plateau and in various, mainly mountainous parts of Hungary (Transdanubian Mountains, Mecsek Mountains, North Hungarian Mountains) (Fig. 2). Study areas included roosting sites localized in caves and mine galleries. Dates for capturing bats ranged from 1998 to 2015.

### Capture of bats and collection of bat flies

Bats were captured mostly close to roosting sites using mist nets or harp traps located at drinking, foraging and swarming sites. All bats were identified [29] and sex and age was determined for each specimen (whenever possible). Ectoparasitic bat flies were removed using forceps. The only exception to this method is the bat flies from Hungarian bats captured in 1998–1999, which were collected with the help of a Fair Isle Apparatus. The Fair Isle Apparatus method was originally developed for collecting ectoparasites of birds [30]. Bats are placed in a small plastic box but keeping their head outside while ethyl acetate is used as fumigant inside the box. After parasite removal, bats were immediately released at the place of capture.

Preservation and long-term storage of bat flies was in 70 or 87% ethanol in separate vials (one vial per bat host). A small number of the Romanian flies were collected from corpses of bats found in caves or early emerging ones (*Myotis daubentonii*, *n* =12, Cheile Turzii, Romania). Identification of bat flies was based on Theodor [31] and Theodor & Moscona [32]. Voucher specimens are deposited at Eszterházy Károly University (Hungary) under accession nos. 12EP01 through 12EP95, 14EP01 through 14EP21, 15EP01 through 15EP07 and P001 through P178. In this study, we collected all ectoparasites from each individual bat to allow prevalence data to be calculated.

### Identification of Laboulbeniales

Bat flies were screened for ectoparasitic fungi using a binocular microscope. Position and density of thalli were detailed and thalli were removed with an entomological pin and slide-mounted for identification [13]. Identification was based on the descriptions and drawings in Thaxter [33]. Voucher slides are deposited at BP (Botanical Department, Hungarian Natural History Museum; nos. 107915 & 107916) and FH (Farlow Herbarium, Harvard University; no. D. Haelew. 1015c) (details in Additional file 2: Table S2).

### DNA extraction from Laboulbeniales, PCR and sequencing

Isolation of Laboulbeniales DNA was carried out using the Extract-N-Amp Plant PCR Kit (Sigma-Aldrich, St Louis, MO, USA) and the heat extraction protocol, as outlined in a previous study [28]. PCR amplification of small subunit (SSU) and large subunit (LSU) ribosomal DNA (rDNA) was performed using the following primer combinations: SL122/NSL2 [28, 34] and NS1/NS4 [35] for SSU, LIC24R/LR3 [36, 37] and LR0R/LR5 [37] for LSU. PCR conditions were: denaturation at 94 °C for 3 min, followed by 35 cycles at 94 °C for 1 min, 50 °C for 45 s, and 72 °C for 1:30 min, and a final extension step of 72 °C for 10 min. In the case of unsuccessful PCR, a modified touchdown protocol was applied: initial denaturation at 95 °C for 10 min, followed by 30 cycles at 95 °C for 1 min, 62 °C for 1 min (decreasing 1 °C every 3 cycles), and 72 °C for 1:30 min; then 30 cycles with denaturation at 95 °C for 30 s, 55 °C for 30 s, and 72 °C for 1 min, and a final extension step of 72 °C for 7 min. PCR amplification of the internal transcribed spacer (ITS) rDNA was attempted, but without success. PCR products were purified using QIAquick PCR purification kit (Qiagen, Stanford, CA, USA), then sequenced as described in Haelewaters et al. [28]. Generated DNA sequences were assembled and edited in Sequencher 4.10.1 (Gene Codes Corporation, Ann Arbor, MI) and blasted in NCBI GenBank (http://ncbi.nlm.nih.gov/blast/Blast.cgi).

The genus *Arthrorhynchus* was positioned within the Stigmatomycetinae subtribe (= Stigmatomyceteae [33, 38]) based on the receptacle structure with three superposed cells [21]. This subtribe holds 39 genera [38, 39], among which *Corethromyces* Thaxt., *Hesperomyces* Thaxt., *Prolixandromyces* R.K. Benj., *Rhadinomyces* Thaxt. and *Stigmatomyces* H. Karst. Sequences of species in these genera are present in GenBank and thus we blasted our SSU rDNA sequences against the following species: *Corethromyces bicolor* (GenBank: AF431762), *Corethromyces* sp. (AF431761), *Hesperomyces coleomegillae* (KF266893), *Hesperomyces virescens* (KU574866), *Prolixandromyces triandrus* (LT158294), *Rhadinomyces pallidus* (AF431763), *Stigmatomyces borealis* (JN835186) and *S. limnophorae* (AF407576). For the LSU rDNA region, much less Laboulbeniales sequences are available in GenBank. We blasted our LSU rDNA sequences against the following species: *Hesperomyces virescens* (KU574867) and *Prolixandromyces triandrus* (LT158295).

### Statistical analysis

Laboulbeniales infection fractions for *Penicillidia conspicua*, *P. dufourii* and *Nycteribia schmidlii* were statistically analyzed using generalized linear mixed models (GLMM), using the R package *lme4* [40]. We took the binomial distribution with logit link function for the binary infection score per bat fly (aggregating the binary scores per bat gave no indication of overdispersion). The GLMM contained fixed effects for the three bat fly species, the sex of the bat fly, and their interaction, and random effects for the location and year of data collection. First, the sex effect on infection fraction was studied per bat fly species, and, if allowed, the sex effects were aggregated over the three species.

Hypothesis testing was done using likelihood ratio tests, with *P*-values calculated based on *χ* ^2^ distributions, declaring an effect significant when *P* < 0.05. Outcomes of the test statistics are reported as *X*_*df*_^2^ with *df* = the number of parameters tested simultaneously.

### Visualization of the host–parasite–parasite network

The bat-bat fly-Laboulbeniales associations were visualized with the help of the R package *bipartite* [41]. We used weighted data and the function *plotweb* to build a network showing host-dependencies and prevalence.

## Results

### Bats and bat flies

We sampled 1,594 bats of 28 species. Of these, 997 bats were captured in Hungary (24 species), of which 361 carried bat flies (parasite prevalence of 36%). In Romania, 597 bats were captured (10 species), of which 186 had bat flies (31%). Taken together, 547 bats were infected with bat flies, accounting for an overall prevalence of 34%. Details are presented in Table 1. Infected bats were found with different numbers of bat flies. The highest number of bat flies harvested from a single bat was 21. This was *Myotis daubentonii* with only *Nycteribia kolenatii* bat flies from Hungary (sample P161). Overall, *M. daubentonii* (220 infected bats) carried the highest number of bat flies, with 22 specimens having ≥ 10 bat flies each. In comparison, also many *Miniopterus schreibersii* bats were infected by bat flies (179), but only one carried ≥ 10 bat flies. Altogether 270 different bat specimens had a single bat fly. Numbers of infected bats are given in Table 2, along with minimum and maximum number of bat flies collected per bat. Of the 28 sampled bat species, 13 were not found to host bat flies (46%). Collection data of all surveyed 1,494 bat fly specimens are listed in Additional file 2: Table S2.

**Table 1:** Overview of studied bats. Number of bats of different species surveyed in this study, along with the number of bat fly-infected individuals and number of collection sites where the bat species was recorded

Bat species	Hungary			Romania		
	No. of bats	No. of bats with bat flies	No. of collection sites	No. of bats	No. of bats with bat flies	No. of collection sites
*Barbastella barbastellus*	56	1	20			
*Eptesicus serotinus*	2	0	2			
*Hypsugo savii*	2	0	2			
*Miniopterus schreibersii*	89	79	4	242	100	5
*Myotis alcathoe*	31	4	17			
*Myotis bechsteinii*	199	38	23			
*Myotis blythii*	14	7	5	16	9	3
*Myotis brandtii*	15	5	7			
*Myotis capaccinii*				9	2	1
*Myotis dasycneme*	15	0	9			
*Myotis daubentonii*	234	183	28	151	37	4
*Myotis emarginatus*	38	0	10			
*Myotis myotis*	33	20	11	47	19	5
*Myotis mystacinus*	1	0	1			
*Myotis nattereri*	91	6	15	9	1	1
*Nyctalus leisleri*	1	0	1			
*Nyctalus noctula*	3	0	2			
*Pipistrellus nathusii*	2	0	2			
*Pipistrellus pipistrellus*	35	0	9			
*Plecotus auritus*	47	4	15			
*Plecotus austriacus*	1	0	1			
*Plecotus sp. indet.*	1	1	1			
*Rhinolophus blasii*				12	3	1
*Rhinolophus euryale*	36	1	6	9	3	2
*Rhinolophus ferrumequinum*	18	11	6	73	5	3
*Rhinolophus hipposideros*	29	1	10			
*Rhinolophus mehelyi*				29	7	2
*Vespertilio murinus*	4	0	3			
Total	997	361		597	186	
Total both countries	1,594	547

**Table 2:** Overview of bats with bat flies. Number of bats with bat flies per country (Hungary, Romania). Per bat species, the minimum and maximum number of bat flies collected from a single bat as well as the average number of bat flies collected per bat species are given

Bat species	No. of bats with bat flies		No. of bat flies on bat hosts		
	Hungary	Romania	Minimum	Maximum	Average
*Barbastella barbastellus*	1	0	1	1	1.00
*Miniopterus schreibersii*	79	100	1	13	1.75
*Myotis alcathoe*	4	0	1	2	1.50
*Myotis bechsteinii*	38	0	1	4	1.37
*Myotis blythii*	7	9	1	4	1.81
*Myotis brandtii*	5	0	1	1	1.00
*Myotis capaccinii*	0	2	2	4	3.00
*Myotis daubentonii*	183	37	1	21	4.13
*Myotis myotis*	20	19	1	16	2.59
*Myotis nattereri*	6	1	1	2	1.14
*Plecotus auritus*	4	0	1	1	1.00
*Plecotus* sp. indet.	1	0	2	2	2.00
*Rhinolophus blasii*	0	3	1	2	1.67
*Rhinolophus euryale*	1	3	1	4	2.00
*Rhinolophus ferrumequinum*	11	5	1	6	2.06
*Rhinolophus hipposideros*	1	0	1	1	1.00
*Rhinolophus mehelyi*	0	7	1	2	1.43
Total bats with bat flies	361	186			

Among the bat fly specimens collected in this study, a new country record for Hungary was found, *Nycteribia pedicularia* Latreille, 1805, collected from *Myotis daubentonii* (20 specimens) and *Myotis myotis* (2 specimens). This fly species was recovered from the sampling localities in Western Hungary (Isztimér, Komló, Mánfa, Olaszfalu, Őcsény and Pogány).

Furthermore, our collections revealed new bat-bat fly associations [42]: *Nycteribia kolenatii* Theodor & Moscona, 1954 is reported for the first time parasitizing *Miniopterus schreibersii*, *Myotis blythii*, *Myotis capaccinii* and *Rhinolophus ferrumequinum*. Similarily, *Penicillidia conspicua* is reported from *Myotis daubentonii* and *Phthiridium biarticulatum* Hermann, 1804 from *M. capaccinii*.

### Laboulbeniales on bat flies

**Fig. 3:**
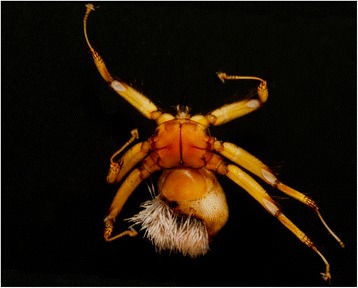
Heavily infected bat fly *Penicillidia conspicua* with many thalli of *Arthrorhynchus nycteribiae* ventrally on its abdomen (sample 101206, Romania)

**Fig. 4:**
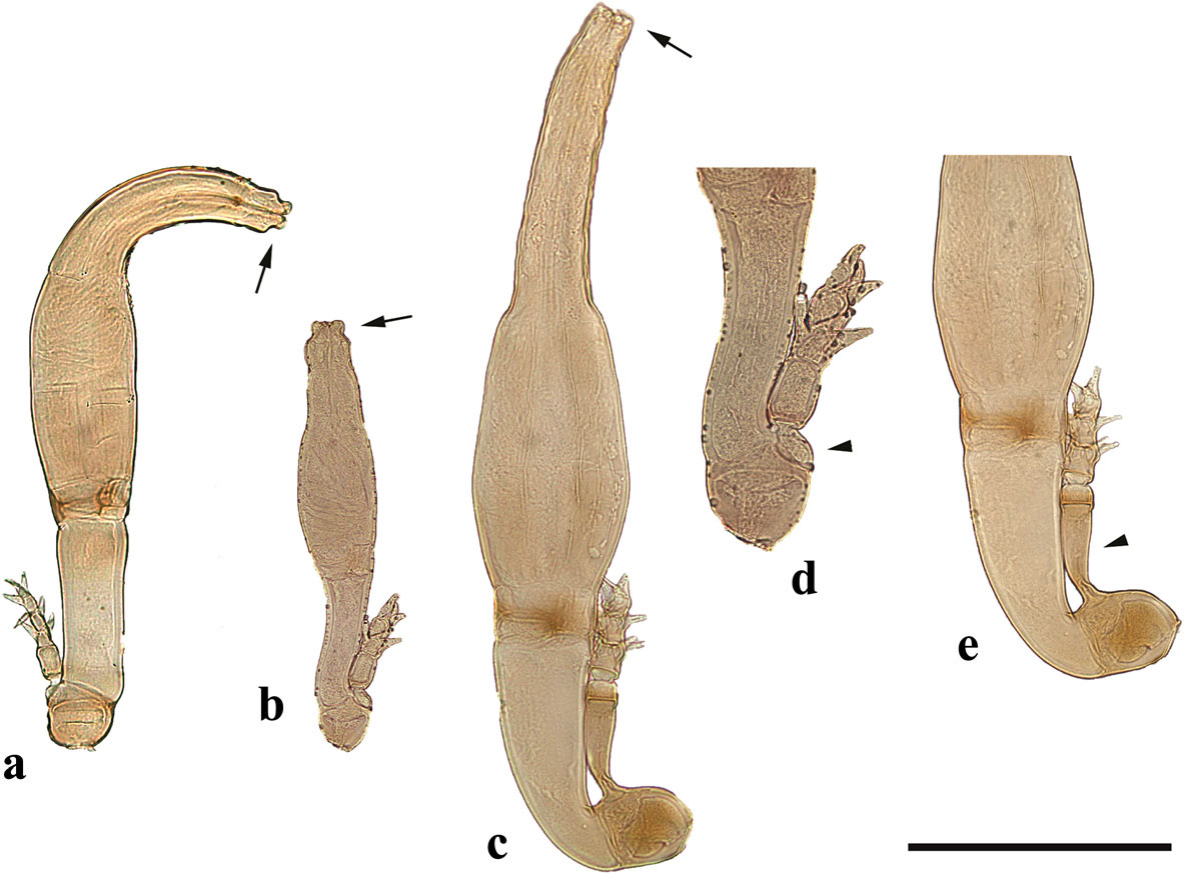
*Arthrorhynchus* species encountered during this study. **a** Single thallus of *Arthrorhynchus eucampsipodae* with curved perithecium (sample 12EP50). **b** Single thallus of *A. eucampsipodae* (sample P052). **c** Single thallus of *Arthrorhynchus nycteribiae* (sample 14EP24). **d** Detail of basal part of *A. eucampsipodae* (sample P052). **e** Detail of basal part of *A. nycteribiae* (sample 14EP24). Both species can be distinguished based on the perithecial tip (arrows in **a**, **b**, and **c**) and cell III of the receptable (arrowheads in **d** and **e**). *Scale-bar*: **a-c**, **e**, 200 μm; **d**, 100 μm

We found infection with Laboulbeniales on 45 of 1,494 bat flies (3%). All Laboulbeniales belonged in the genus *Arthrorhynchus*. In Romania, we recorded *A. nycteribiae* on *Penicillidia conspicua* (16 specimens) (Fig. 3) and *P. dufourii* (2 specimens). In Hungary, we found two species of bat fly-associated Laboulbeniales: *A. eucampsipodae* (Fig. 4a-b, d), a new country record, and *A. nycteribiae* (Fig. 4c, e): *Arthrorhynchus eucampsipodae* was recorded on *Nycteribia schmidlii* (4 specimens), while *A. nycteribiae* was found on *P. conspicua* (22 specimens) and *N. schmidlii* (1 specimen). Data on the prevalence of Laboulbeniales infection among all bat fly species are presented in Table 3.

**Table 3:** Overview of studied bat flies. Bat fly species sampled from Hungary and Romania during this study, with the prevalence of Laboulbeniales infections and indication of parasite species

Bat fly species	Bat host	Country	No. sampled	No. infected	% infected	Country	Laboulbeniales species
*Basilia italica*	All	H	13	0			
*Basilia nana*	All	H	49	0			
*Basilia nattereri*	All	H	16	0			
*Nycteribia kolenatii*	All	H, RO	914	0			
*Nycteribia latreillii*	All	H	3	0			
*Nycteribia pedicularia*	All	H	22	0			
*Nycteribia schmidlii*	*Miniopterus schreibersii*	H, RO	147	5	3.1	H	*Arthrorhynchus eucampsipodae* (4) *Arthrorhynchus nycteribiae* (1)
*Nycteribia schmidlii*	Other bat host species	H, RO	12	0			
*Nycteribia vexata*	All	H, RO	14	0			
*Penicillidia conspicua*	*Miniopterus schreibersii*	H, RO	142	33	23.1	H, RO	*Arthrorhynchus nycteribiae*
*Penicillidia conspicua*	*Myotis daubentonii*	RO	7	4	57.1	RO	*Arthrorhynchus nycteribiae*
*Penicillidia conspicua*	*Myotis blythii*	RO	2	0			
*Penicillidia conspicua*	*Rhinolophus euryale*	H	1	1	100	H	*Arthrorhynchus nycteribiae*
*Penicillidia dufourii*	*Myotis myotis*	H, RO	51	2	3.9	RO	*Arthrorhynchus nycteribiae*
*Penicillidia dufourii*	Other bat host species	H, RO	51	0			
*Phthiridium biarticulatum*	All	H, RO	50	0			
Total			1,494	45	3.0		

*Abbreviations*: *H* Hungary, *RO* Romania

Four new sequences determined in this study are deposited in GenBank: *Arthrorhynchus nycteribiae* from *Penicillidia conspicua* on *Rhinolophus euryale* (Hungary, Edelény), 14EP24, SSU KY094496/ LSU KY094497; and *A. nycteribiae* from *P. conspicua* on *Miniopterus schreibersii* (Hungary, Felsőtárkány), 12EP144, isolate D. Haelew. 1015d, SSU KY094498/ LSU KY094499. We blasted our longest SSU (1,075 bp) and LSU rDNA (877 bp) sequences against selected species, listed in Table 4. The similarity ranged between 87 and 94% for SSU, and between 85 and 89% for LSU.

**Table 4:** Blast search results for *Arthrorhynchus nycteribiae* SSU and LSU rDNA sequences (isolate from Edelény). To confirm the accuracy of these newly generated sequences a second isolate was sequenced, from another locality (Felsőtárkány) and another bat host. The first row shows the blast results of the two *A. nycteribiae* isolates against each other

Species	SSU	Isolate	SSU blast (%)	LSU	Isolate	LSU blast (%)
*Arthrorhynchus nycteribiae*	KY094498	D. Haelew. 1015d	99	KY094499	D. Haelew. 1015d	100
*Corethromyces bicolor*	AF431762	–	?			
*Corethromyces* sp. AW-2001	AF431761	–	88			
*Hesperomyces coleomegillae*	KF266893	voucher 637	94			
*Hesperomyces virescens*	KU574866	D. Haelew. 655c	94	KU574867	D. Haelew. 655c	85
*Prolixandromyces triandrus*	LT158294	Nagyvisnyo1	94	LT158295	Nagyvisnyo1	89
*Rhadinomyces pallidus*	AF431763	–	88			
*Stigmatomyces borealis*	JN835186	AW-797	87			
*Stigmatomyces limnophorae*	AF407576	–	89			

**Fig. 5:**
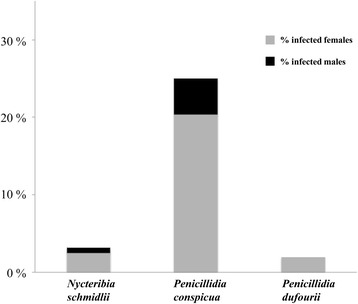
Relationship between bat fly sex and infection with Laboulbeniales. In all three bat fly species with Laboulbeniales, female infected bat flies are more frequently (or the only ones) encountered. Of 5 infected *Nycteribia schmidlii* flies (*n* = 159), 4 were female. Of 38 infected *Penicillidia conspicua* flies (*n* = 152), 31 were female. And both infected *Penicillidia dufourii* flies (*n* = 102) were female

Of the 159 sampled *N. schmidlii* bat flies, 5 were infected with Laboulbeniales (3.1%). Four infected flies were females, 1 male. For *P. conspicua*, 38 of 152 bat flies were infected with Laboulbeniales (25%). Of these infected flies 31 were female and only 7 were male. For *P. dufourii*, 2 of 102 bat flies were infected with Laboulbeniales (2.0%), both females. (Fig. 5).

Although in all three bat fly species the female infection fractions were higher, in none of them a significant difference between infection fractions of female and male bat flies was found (GLMM: *P. conspicua*: *X*_1_^2^ = 3.19, *P* = 0.074; *P. dufourii*: *X*_1_^2^ = 2.52, *P* = 0.113; *N. schmidlii*: *X*_1_^2^ = 0.21, *P* = 0.648). The interaction between bat fly species and sex was not significant (GLMM: *X*_2_^2^ = 1.35, *P* = 0.510). We therefore removed this interaction from the model. In the resulting GLMM we found a significant difference in infection rate between female and male bat flies (averaged over species; *X*_1_^2^ = 4.56, *P* = 0.0327, higher in females), and highly significant differences in infection rates among bat fly species (averaged over sexes; *X*_2_^2^ = 52.83, *P* < 0.0001). No extra variation due to year of data collection was found, but variation due to location of data collection was considerable *X*_1_^2^ = 12.12 (*P* = 0.00025, obtained by halving the *P*-value from the *χ*_1_^2^ distribution).

Infected *P. conspicua* bat flies were sampled from three different bat hosts: *Myotis daubentoni* (4 infected bat flies, *n* = 7); *Miniopterus schreibersii* (33, *n* = 142); and *Rhinolophus euryale* (1, *n* = 1). Infected *P. dufourii* bat flies were found only on the bat *Myotis myotis* and infected *N. schmidlii* only on the host *Miniopterus schreibersii*. A detailed summary, including collecting data, is presented in Additional file 3: Table S3.

### Host-parasite-parasite network

**Fig. 6:**
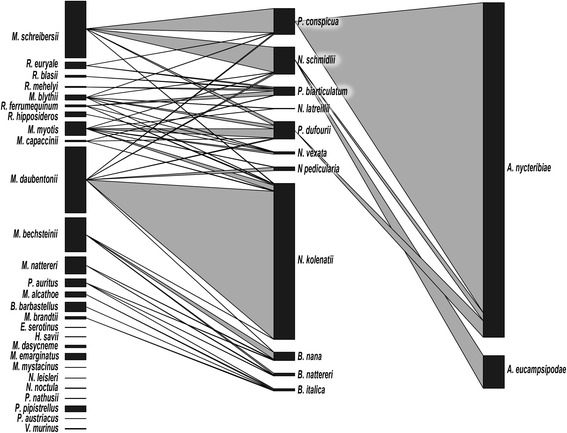
Host-parasite-parasite network. Shown is the association of bat flies with their bat host as well as the association of Laboulbeniales and their arthropod hosts. The width of the bars represents the relative abundance of a single species within each network level

Figure 6 shows the association of bat flies with their bat host as well as the association of Laboulbeniales and their arthropod hosts. The principal bat host for bat flies infected with Laboulbeniales species was *M. schreibersii* for *P. conspicua* and *N. schmidlii. Penicillidia dufourii* was found mainly on *M. myotis* but also occurred commonly on *M. blythii* and *M. schreibersii*.

## Discussion

### Distribution and associations of bat flies and their bat hosts

Bat flies generally have one or two preferred bat hosts upon which they are collected with higher probability compared to other hosts [31, 42]. Our dataset supports these main hosts for the bat flies with a few notable deviations. In the case of *Basilia nana* we found most flies on *Myotis bechsteinii* (*n* = 41), one of the two major hosts, and fewer individuals from the other host, *M. nattereri* (*n* = 4). *Phthiridium biarticulatum* has two major hosts, and we mostly found them on *Rhinolophus ferrumequinum* (*n* = 26), while the other host species, *R. hipposideros*, is practically missing from our dataset. The new country record for Hungary, *Nycteribia pedicularia*, has *Myotis capaccinii* as its main bat host species, but we found it on *M. daubentonii* (*n* = 20) and *M. myotis* (*n* = 2).

The new host associations in case of the bat flies *Nycteribia kolenatii*, *Penicillidia conspicua* and *Phthiridium biarticulatum* only extend the list of known hosts, since our collecting records support the existing major host species.

### Morphological identification of *Arthrorhynchus*

In our circumscription of the genus, three species are accepted: *A. cyclopodiae*, *A. eucampsipodae* and *A. nycteribiae*. The three species can be distinguished based on two morphological characteristics: cell III and the perithecial tip (Fig. 4). *Arthrorhynchus nycteribiae* is easily separated from the other two species by the elongated cell III, which carries the appendage ([33]: Plate XLVIII, 7–9). In addition, its perithecial tip is four-lobed, with each of the four lobes conspicuously three-lobed ([33]: Plate XLVIII, 10). The perithecial tip of *A. eucampsipodae* is very similar to that of *A. nycteribiae* but the four lobes are not individually three-lobed. The perithecial tip in *A. cyclopodiae* also ends in four lobes but these are not short and broad as in *A. eucampsipodae* and *A. nycteribiae*, but erect. *Arthrorhynchus cyclopodiae* and *A. eucampsipodae* have similar appendage structures, however there are some differences. In *A. eucampsipodae*, cell III is constricted in the middle, the upper part of it being conspicuously smaller compared to the lower half; it is also narrower than the basal cell of the appendage ([33]: Plate XLVIII, 14). Cell III of *A. cyclopodiae* is also constricted in the middle but the upper half is inflated and mostly broader than the base of the basal cell of the appendage ([33]: Plate XLVIII, 3).

Variation in the thallus morphology of *A. eucampsipodae* was noted by Thaxter [33], who mentioned that its perithecia may be straight or slightly curved distally. Thalli from the locality Szársomlyó (Fig. 4b) showed straight perithecia, although the other half, those collected in Felsőtárkány (Fig. 4a) showed markedly curved (and somewhat longer) perithecia. Both populations were identical in the cell III and the appendage structure.

### DNA sequence data of bat fly-associated Laboulbeniales

Morphological identification may be insufficient to assess the diversity of Laboulbeniales on (temperate) bat flies. There is the phenomenon of position specificity in Laboulbeniales, in which multiple morphotypes of the same species occupy limited portions of the host’s integument [43]. More generally, many fungal species are cryptic, with lineages “hidden” within morphological species complexes. This cryptic diversity is rapidly being uncovered by applying a molecular phylogenetic approach to taxonomic studies [44–46]. In Laboulbeniales, cryptic diversity is a subject of current research, although advances in this field have been hindered by the difficulties in working with and extracting DNA from these fungi [28]. Preservation of material is of utmost importance in successfully extracting DNA from Laboulbeniales: freshly collected host specimens preferably stored in ≥ 95% ethanol give the best results [28]. To fully understand and discuss the diversity of Laboulbeniales on specific host groups, we need to generate DNA sequences of as many species as we encounter, to be able to add a phylogenetic component to morphological data.

For this study, we generated the first DNA sequences of *A. nycteribiae*. We generated sequences for two isolates. These isolates were made from bat flies from different bat hosts collected in different locations. Additionally, we applied two distinct DNA isolation methods in two of the collaborating laboratories. The SSU sequences match for 99% (over 522 bp) and the LSU sequences are 100% alike (over 450 bp). This confirms molecular identity of our isolates. Blasting the SSU rDNA sequence against several species in the Stigmatomycetinae shows that *Hesperomyces* and *Prolixandromyces* are more closely related to *Arthrorhynchus*, compared to *Corethromyces*, *Rhadinomyces* and *Stigmatomyces*. No significant similarity was found with *C. bicolor*.

Traditionally, the Laboulbeniales (as well as the class Laboulbeniomycetes) are excluded from major phylogenies of the Ascomycota. This is due to a lack of sequence data and the difficulty of working with these fungi . Currently, no broadly inclusive phylogeny of the order is available because of the lack of adequate sampling. However, preliminary data suggest that the Stigmatomycetinae are not a monophyletic group (D. Haelewaters, unpublished data). Generating more DNA sequences and increasing taxon sampling will be important to the resolution of relationships within the Laboulbeniales and to better understand how species have evolved and developed some of their unique traits (such as the formation of a thallus). As to bat fly-associated Laboulbeniales, determining the position of the genera *Arthrorhynchus*, *Gloeandromyces* and *Nycteromyces* within the phylogeny of the order may reveal that they form a single clade or, on the contrary, that multiple colonizations on bat flies from other host groups have happened with subsequent diversification *sensu* De Kesel & Haelewaters [47].

The number of thalli of *A. eucampsipodae* was insufficient for molecular work. We will continue to sample fresh bat flies and screen for *Arthrorhynchus* spp. from different populations of different host species. We hope this will lead to more sequences. It will be a difficult endeavor to recollect *A. cyclopodiae*, since it is currently only known from the type locality. However, comparing isolates of *A. nycteribiae* taken from different populations of the same host species and from different host species, will give a good estimate of the diversity of Laboulbeniales in this temperate system.

### Diversity of bat fly-associated Laboulbeniales in central Europe

Laboulbeniales exhibit three types of specialization: high specificity to host species (host specificity), growth restricted to certain areas of the host body (position specificity), and speciation resulting from co-habiting hosts (ecological specificity) [43, 47–49].

*Arthrorhynchus eucampsipodae* is known from species of *Basilia*, *Cyclopodia*, *Eucampsipoda*, *Nycteribia* and *Penicillidia* bat flies (Additional file 1: Table S1). *Arthrorhynchus nycteribiae* has been found on species of *Nycteribia*, *Penicillidia* and *Phthiridium*. In our study, we found only *Nycteribia schmidlii*, *Penicillidia conspicua* and *P. dufourii* infected by *Arthrorhynchus eucampsipodae* and *A. nycteribiae*. These three species, together with *Phthiridium biarticulatum*, are most commonly encountered with Laboulbeniales [21]. Except for being seemingly restricted to Eastern Hemisphere species of Nycteribiidae, there is no strict host specificity, as previously reported [21].

*Nycteribia schmidlii* was host for both *A. eucampsipodae* and *A. nycteribiae* in our study. No double infection was found and both fungi were sampled from bat flies from different populations of *M. schreibersii* in Hungary (Additional file 2: Table S2): infection with *A. eucampsipodae* in Felsőtárkány and Szársomlyó, infection with *A. nycteribiae* in Nagyharsány. Thus far, no double infections have been reported from a single bat fly in the Eastern Hemisphere. This is contrary to the Western Hemisphere, where double infections are observed regularly. Thaxter [22, 50] reported a double infection of *Gloeandromyces streblae* and *Nycteromyces streblidinus* on *Strebla wiedemanni*. More recently, we have detected double infections of *G. streblae* and *Gloeandromyces* n. spp. on various streblid bat flies from Ecuador, Nicaragua and Panama (D. Haelewaters et al., unpublished data). Physical contact (e.g. mating) between bat flies hosting mature thalli of different *Arthrorhynchus* species could lead to cross-infection and the presence of double infections on hosts. The main reason for the apparent absence of double infections may simply be the rarity of infected flies, and hence, of contacts between two differently infected flies.

Do bat flies sharing the same bat host carry the same species of Laboulbeniales? If they do share hosts, this would represent an example of ecological specificity. In this situation, the bat skin/fur itself acts as a microhabitat. Also caves, ant nests, and fragmented habitats in salt marshes can be cohabited by multiple, often unrelated hosts [47, 49, 51, 52]. In our dataset, we found three bat fly hosts for *A. nycteribiae*. These are *Penicillidia conspicua*, *P. dufourii* and *Nycteribia schmidlii*. The fungus was present on two specimens of *P. dufourii* and on a single specimen of *N. schmidlii*, while it was associated with 37 specimens of *P. conspicua*. It seems that *P. dufourii* and *N. schmidlii* are “accidental hosts” and *P. conspicua* the “main host”. *Penicillidia dufourii* uses *Myotis myotis* and *M. blythii* but it is also found on *Miniopterus schreibersii* (20% in our material). This is probably due to the roost sharing habits of these three bat species; they frequently form mixed colonies in caves [53]. *Miniopterus schreibersii* is the main bat host for *N. schmidlii* and *P. conspicua*. Furthermore, *P. conspicua* and *P. dufourii* sometimes choose non-primary hosts if the opportunity arises [54]. Four *P. conspicua* bat flies and one specimen of *N. schmidlii* from the same *Miniopterus schreibersii* bat were found infected with *A. nycteribiae*. Given the record of *A. nycteribiae* on *N. schmidlii* is the only one known in the literature (also not mentioned in [21]), we can safely assume that this infection was the result of an accidental transmission. Indeed, in this occasion the bat host served as the microhabitat allowing transmission of ascospores between hosts. In some cases, shifting between co-occurring hosts can lead to adaptation and eventually to speciation [47]. However, “successful colonization of a new host is probably a rare event” [51] as seems the case with Eastern Hemisphere bat flies.

Our dataset of ~1,500 bat flies from 15 different bat host species, different habitat types, and geographic locations from Western Hungary to Eastern Romania allows us to speculate on the diversity of Laboulbeniales associated with bat flies in central Europe. We think that the potential for undiscovered species of Laboulbeniales on central European bat flies is very low, and expected only on rarely collected (in this study) bat fly species. Our study revealed no fungi other than *A. eucampsipodae* and *A. nycteribiae* after screening 914 *Nycteribia kolenatii*, 159 *N. schmidlii*, and 152 *P. conspicua* specimens. Also Blackwell [21], after screening 2,937 bat fly specimens, did not make any note of undescribed diversity of *Arthrorhynchus*.

### Unbalanced sex ratios of infection

Generally, female and juvenile bats are more heavily infected by ectoparasites compared to males [3, 55–60]. Furthermore, pregnant individuals are more parasitized by bat flies than non-pregnant females [57]. Dick and Patterson [61] found significantly more male than female bat flies on Venezuelan bats (>36,500 bat flies included in the survey). This phenomenon was potentially explained by selective host grooming, which removes and/or kills the larger females.

In our dataset, we see a clear preference of Laboulbeniales infections on female bat flies. Thus far in Laboulbeniales, sex-related infection patterns are the direct result of mating behavior of the host. For example, in summertime, *Hesperomyces virescens* Thaxt. occurs mainly at the dorsoposterior of females and the ventroposterior of males [62–64]. Other examples are *Cantharomyces denigratus* Thaxt./*C. italicus* Speg. [65], *Chitonomyces* spp. [43] and *Monoicomyces matthiatis* T. Majewski [66].

For bat fly-associated Laboulbeniales other factors seem at play. First, female bat flies live longer than males and have an average life span of about 5–6 weeks [67]. Laboratory studies of *Basilia hispida* Theodor, 1967 bat flies (Nycteribiidae) revealed that males lived for at least 97 days, while females lived for at least 156 days [67]. It could be that the fungal parasites need this time for successful development, maturation, and build-up of inoculum. Second, during pregnancy, female bat flies are significantly larger than males—at extrusion, prepupae may comprise about one third of the body size of the female fly. Moreover, a descriptive modeling study on tsetse flies (Glossinidae: *Glossina* spp.), which belong to the same superfamily as bat flies (Hippoboscoidea), demonstrated that during each pregnancy females accumulate an excess of fat reserves [68], which are manifested as lobes in the haemolymph for maximal exposure [69, 70]. Such reserves represent higher nutritional resources in female flies for parasites such as Laboulbeniales relative to males. A combination of these factors may lead to a greater infection prevalence on female bat flies, as we have observed.

## Conclusions

Our knowledge about Laboulbeniales fungal ectoparasites of bat flies is poor. Seven species in three genera are recognized based on morphological descriptions, although one species is doubtful. Of those seven, four species are only known from the type collections, which are between 65 and 116 years old. For this paper, we collected bat flies from captured bats in central Europe (Hungary, Romania) and screened them for presence of Laboulbeniales. Our survey shows a complex network of bats, bat flies and Laboulbeniales. New bat-bat fly associations are reported: *Nycteribia kolenatii* on *Miniopterus schreibersii*, *Myotis blythii*, *Myotis capaccinii* and *Rhinolophus ferrumequinum*; *Penicillidia conspicua* on *Myotis daubentonii*; and *Phthiridium biarticulatum* on *Myotis capaccinii*. While the bat flies were relatively abundant and diverse (studied material), the Laboulbeniales associated with them were rare and species-poor. Laboulbeniales were found on 45 of 1,494 screened bat flies: *Arthrorhynchus eucampsipodae* was reported on *Nycteribia schmidlii*, and *A. nycteribiae* on *N. schmidlii*, *Penicillidia conspicua* and *P. dufourii. Penicillidia conspicua* was infected most often, followed by *N. schmidlii* and *P. dufourii*. The bat fly *Nycteribia pedicularia* and the fungus *Arthrorhynchus eucampsipodae* represent new country records for Hungary. We generated SSU and LSU ribosomal DNA sequences for *A. nycteribiae*. These are the first sequences for any species of bat fly-associated Laboulbeniales.

## Additional files

Additional file 1: Table S1. Summary of all records of Laboulbeniales associated with bat flies thus far. (XLS 53 kb)
Additional file 2: Table S2. Collection data of all 1,494 bat fly specimens sampled in this study and screened for presence of Laboulbeniales. Infected bat flies are highlighted and documentation is provided about the identification of the fungus and the position(s) on which thalli were found. (XLS 395 kb)
Additional file 3: Table S3. Data for the 45 bat flies infected with Laboulbeniales fungi encountered during this study. Given are bat and bat fly species, sex of bat and bat fly (F = female, M = male), country (H = Hungary, RO = Romania) and locality, year of collection, and position of infection on the bat fly host. (DOC 86 kb)

## Abbreviations

BP: Unique identifier for the herbarium at the Botanical Department, Hungarian Natural History Museum (consistent with Index Herbariorum)
FH: Unique identifier for the Farlow Herbarium at Harvard University (consistent with Index Herbariorum)
GLMM: Generalized linear mixed models
ITS: The internal transcribed spacer region of ribosomal DNA
LSU: The large subunit of ribosomal DNA
PCR: Polymerase chain reaction
rDNA: Ribosomal DNA
SSU: The small subunit of ribosomal DNA
