# Supplementary material for: Parasites of parasites of bats: Laboulbeniales (Fungi: Ascomycota) on bat flies (Diptera: Nycteribiidae) in central Europe
Source: Parasit Vectors. 2017 Feb 21;10:96. doi: 10.1186/s13071-017-2022-y (PMC5320862; doi:10.1186/s13071-017-2022-y)
Supplement: Additional file 3: Table S3. — Data for the 45 bat flies infected with Laboulbeniales fungi encountered during this study. Given are bat and bat fly species, sex of bat and bat fly (F = female, M = male), country (H = Hungary, RO = Romania) and locality, year of collection, and position of infection on the bat fly host. (DOC 86 kb) [file 13071_2017_2022_MOESM3_ESM.doc]

**Additional file 3: Table S3.** Data for the 45 bat flies infected with Laboulbeniales fungi encountered during this study. Given are bat and bat fly species, sex of bat and bat fly (F = female, M = male), country (H = Hungary, RO = Romania) and locality, year of collection, and position of infection on the bat fly host

| **Bat species** | **Bat sex** | **Bat fly** | **Bat fly sex** | **Laboulbeniales** | **Country** | **Location** | **Year** | **Position(s) infected** |
| --- | --- | --- | --- | --- | --- | --- | --- | --- |
| *Miniopterus schreibersii* | F | *Nycteribia schmidlii* | F | *Arthrorhynchus eucampsipodae* | H | Szársomlyó | 1998 | abdomen, dorsal |
| *Miniopterus schreibersii* | F | *Nycteribia schmidlii* | F | *Arthrorhynchus eucampsipodae* | H | Szársomlyó | 1998 | abdomen, dorsal |
| *Miniopterus schreibersii* | F | *Nycteribia schmidlii* | F | *Arthrorhynchus eucampsipodae* | H | Felsőtárkány | 2012 | abdomen, dorsal |
| *Miniopterus schreibersii* | - | *Nycteribia schmidlii* | M | *Arthrorhynchus eucampsipodae* | H | Felsőtárkány | 2015 | abdomen, dorsal |
| *Miniopterus schreibersii* | M | *Penicillidia conspicua* | F | *Arthrorhynchus nycteribiae* | H | Nagyharsány | 2009 | abdomen |
| *Miniopterus schreibersii* | M | *Penicillidia conspicua* | F | *Arthrorhynchus nycteribiae* | H | Nagyharsány | 2009 | abdomen |
| *Miniopterus schreibersii* | M | *Penicillidia conspicua* | F | *Arthrorhynchus nycteribiae* | H | Nagyharsány | 2011 | genitalia |
| *Miniopterus schreibersii* | M | *Penicillidia conspicua* | F | *Arthrorhynchus nycteribiae* | H | Mánfa | 2012 | abdomen |
| *Miniopterus schreibersii* | M | *Nycteribia schmidlii* | F | *Arthrorhynchus nycteribiae* | H | Nagyharsány | 2012 | abdomen |
| *Miniopterus schreibersii* | F | *Penicillidia conspicua* | F | *Arthrorhynchus nycteribiae* | H | Nagyharsány | 2012 | abdomen |
| *Miniopterus schreibersii* | M | *Penicillidia conspicua* | F | *Arthrorhynchus nycteribiae* | H | Nagyharsány | 2012 | abdomen |
| *Miniopterus schreibersii* | M | *Penicillidia conspicua* | F | *Arthrorhynchus nycteribiae* | H | Nagyharsány | 2012 | abdomen |
| *Miniopterus schreibersii* | M | *Penicillidia conspicua* | F | *Arthrorhynchus nycteribiae* | H | Nagyharsány | 2012 | abdomen |
| *Miniopterus schreibersii* | F | *Penicillidia conspicua* | M | *Arthrorhynchus nycteribiae* | H | Felsőtárkány | 2014 |  |
| *Miniopterus schreibersii* | - | *Penicillidia conspicua* | M | *Arthrorhynchus nycteribiae* | H | Felsőtárkány | 2015 |  |
| *Miniopterus schreibersii* | - | *Penicillidia conspicua* | M | *Arthrorhynchus nycteribiae* | H | Felsőtárkány | 2015 |  |
| *Miniopterus schreibersii* | F | *Penicillidia conspicua* | F | *Arthrorhynchus nycteribiae* | H | Felsőtárkány | 2012 |  |
| *Miniopterus schreibersii* | M | *Penicillidia conspicua* | F | *Arthrorhynchus nycteribiae* | H | Felsőtárkány | 2012 | abdomen |
| *Miniopterus schreibersii* | M | *Penicillidia conspicua* | F | *Arthrorhynchus nycteribiae* | H | Felsőtárkány | 2012 |  |
| *Miniopterus schreibersii* | M | *Penicillidia conspicua* | F | *Arthrorhynchus nycteribiae* | H | Felsőtárkány | 2012 |  |
| *Miniopterus schreibersii* | F | *Penicillidia conspicua* | F | *Arthrorhynchus nycteribiae* | H | Miskolc | 2012 |  |
| *Miniopterus schreibersii* | M | *Penicillidia conspicua* | F | *Arthrorhynchus nycteribiae* | H | Felsőtárkány | 2012 |  |
| *Miniopterus schreibersii* | F | *Penicillidia conspicua* | F | *Arthrorhynchus nycteribiae* | H | Felsőtárkány | 2013 |  |
| *Miniopterus schreibersii* | F | *Penicillidia conspicua* | F | *Arthrorhynchus nycteribiae* | H | Felsőtárkány | 2015 | abdomen |
| *Miniopterus schreibersii* | - | *Penicillidia conspicua* | F | *Arthrorhynchus nycteribiae* | H | Felsőtárkány | 2015 |  |
| *Miniopterus schreibersii* | - | *Penicillidia conspicua* | F | *Arthrorhynchus nycteribiae* | H | Felsőtárkány | 2015 |  |
| *Myotis daubentoni* | F | *Penicillidia conspicua* | M | *Arthrorhynchus nycteribiae* | RO | Cheile Turzii | 2015 | abdomen |
| *Myotis daubentoni* | F | *Penicillidia conspicua* | M | *Arthrorhynchus nycteribiae* | RO | Cheile Turzii | 2015 | abdomen |
| *Myotis daubentoni* | M | *Penicillidia conspicua* | F | *Arthrorhynchus nycteribiae* | RO | Cheile Turzii | 2015 | abdomen |
| *Myotis daubentoni* | M | *Penicillidia conspicua* | F | *Arthrorhynchus nycteribiae* | RO | Cheile Turzii | 2015 | abdomen and legs |
| *Miniopterus schreibersii* | F | *Penicillidia conspicua* | M | *Arthrorhynchus nycteribiae* | RO | Tulcea | 2015 | abdomen |
| *Miniopterus schreibersii* | F | *Penicillidia conspicua* | F | *Arthrorhynchus nycteribiae* | RO | Tulcea | 2015 | abdomen |
| *Miniopterus schreibersii* | M | *Penicillidia conspicua* | F | *Arthrorhynchus nycteribiae* | RO | Tulcea | 2015 | abdomen |
| *Miniopterus schreibersii* | M | *Penicillidia conspicua* | M | *Arthrorhynchus nycteribiae* | RO | Betfia | 2015 |  |
| *Miniopterus schreibersii* | M | *Penicillidia conspicua* | F | *Arthrorhynchus nycteribiae* | RO | Betfia | 2015 |  |
| *Miniopterus schreibersii* | M | *Penicillidia conspicua* | F | *Arthrorhynchus nycteribiae* | RO | Betfia | 2015 | abdomen, palp, legs |
| *Miniopterus schreibersii* | M | *Penicillidia conspicua* | F | *Arthrorhynchus nycteribiae* | RO | Tulcea | 2015 | abdomen |
| *Miniopterus schreibersii* | M | *Penicillidia conspicua* | F | *Arthrorhynchus nycteribiae* | RO | Tulcea | 2015 | abdomen |
| *Miniopterus schreibersii* | M | *Penicillidia conspicua* | F | *Arthrorhynchus nycteribiae* | RO | Tulcea | 2015 | abdomen |
| *Miniopterus schreibersii* | M | *Penicillidia conspicua* | F | *Arthrorhynchus nycteribiae* | RO | Gura Dobrogei | 2015 | abdomen |
| *Miniopterus schreibersii* | M | *Penicillidia conspicua* | F | *Arthrorhynchus nycteribiae* | RO | Gura Dobrogei | 2015 | abdomen |
| *Miniopterus schreibersii* | F | *Penicillidia conspicua* | F | *Arthrorhynchus nycteribiae* | RO | Gura Dobrogei | 2015 | ovipositor |
| *Myotis myotis* | F | *Penicillidia dufourii* | F | *Arthrorhynchus nycteribiae* | RO | Betfia | 2015 | head |
| *Myotis myotis* | F | *Penicillidia dufourii* | F | *Arthrorhynchus nycteribiae* | RO | Betfia | 2015 | head |
| *Rhinolophus euryale* | - | *Penicillidia conspicua* | F | *Arthrorhynchus nycteribiae* | H | Edelény | 2014 | abdomen |
